# Cytotoxic Effects of Cnidarian Venoms: One Cancer, Two Cell Lines

**DOI:** 10.1007/s10126-026-10625-2

**Published:** 2026-05-14

**Authors:** Khouloud Azaiez, Nurçin Killi, Kemal Emir Türk, Hazal Kalsın Demir, Anara Babayeva, Esin Sakallı Çetin

**Affiliations:** 1 Graduate School of Natural and Applied Sciences, Fisheries Engineering Program, Muğla Sıtkı Koçman University, Muğla, Türkiye; 2 Faculty of Fisheries, Department of Basic Sciences, Muğla Sıtkı Koçman University, Muğla, Türkiye; 3 Institute of Health Sciences, Medical Biology Program and Faculty of Medicine, Department of Medical Biology, Muğla Sıtkı Koçman University, Muğla, Türkiye; 4 Biotechnology Research, ALM Research Building, Muğla Sıtkı Koçman University, Muğla, Türkiye; 5 Faculty of Medicine, Department of Medical Biology, Muğla Sıtkı Koçman University, Muğla, Türkiye

**Keywords:** *Rhopilema nomadica*, *Aurelia aurita*, *Anemonia sulcata*, Colon cancer, HT29, Caco2

## Abstract

This study aimed to evaluate the cytotoxic effects of jellyfish venoms on two different colon cancer cell lines and to compare their activity with that observed in a healthy colon cell line used as a control. Venoms were isolated from specimens of *Aurelia aurita*, *Rhopilema nomadica*, and *Anemonia sulcata* collected in their natural habitats. These venoms were applied to HT29 and Caco2 colon cancer cell lines, as well as to the CCD18co healthy colon cell line cultured in vitro. The IC₅₀ values of the venoms were determined using the MTT assay. Given the IC₅₀ results, *A. aurita* displayed the highest cytotoxic potency, *R. nomadica* showed a moderate and cell line–dependent effect, and *A. sulcata* exhibited a delayed, high-dose cytotoxicity profile, becoming clearly evident at 72 h. Based on these results, Annexin-V, ROS, and MMP analyses were performed to investigate the mechanisms of cell death. Our findings showed that *A. aurita* venom was more effective on HT29 cells after 48 h and caused minimal damage to CCD18co healthy cells. *A. sulcata* venom exhibited cytotoxic effects on HT29 cells at both 48 and 72 h, while exerting limited toxicity on healthy CCD18co cells. *R. nomadica* venom showed cytotoxicity on Caco2 cells at 48 h. Among the three venoms, *R. nomadica* exhibited the highest cytotoxic effect on CCD18co healthy colon cells. In conclusion, venoms from all three Cnidarian species demonstrated cytotoxic effects on two different colon cancer cell lines. The cytotoxicity was found to be apoptosis-related. Moreover, the venoms showed selective activity across different cancer cell lines.

## Introduction

Paracelsus said, “*Every substance is a poison; no substance is not a poison. The right dose is what distinguishes a poison from a remedy*.” Animal venoms are complex mixtures, the structures of which depend on the species producing venom. The most studied venomous land animals are snakes, scorpions and spiders. Among the marine species, jellyfish, anemones and cone snails are the groups which are the most working on. On the other hand, the venoms have unique biological compounds that can be used in basic sciences and clinical applications. Animal venoms, with their diverse biological functions and a wide range of peptides and proteins, are becoming valuable sources of new compounds that can be used both in basic research and in the development of new drugs (Utkin 2015). In this respect, the aims of our study are below:

1. Determining the cytotoxic effects of venoms of two jellyfish and an anemone species on two different colon cancer lines and comparing these effects.
2. Revealing the cytotoxic activities of Cnidarian venoms on healthy colon cell lines as the control group.
3. Determining the venom effect potential of *Rhopilema nomadica*, an invasive Lessepsian migrant species, compared to natural species and adding a new alternative use possibility to invasive jellyfish.

This is the first study to have been conducted on two different lines of a cancer type and with venom from three different species.

Cnidarians are prominent creatures that have a special organel called “Nematocyst”. A venom which is produced by Golgi apparatus inside in the nematocyst that is used for defense and hunting (Jouiaei et al. 2015). Nematocysts stick like an arrow or coil to prey and transfer the venom. It was known that different nematocyst types have different prey-capturing strategies and different venom compositions (Peach and Pitt 2005). The complex structure of the venom includes enzymes, neurotoxins and species unique peptides. Particularly, phospholipases and metalloproteinases are the major components of venom (Jouiaei et al. 2015). Contact with Cnidaria species can produce effects ranging from mild skin irritation to severe systemic symptoms. Cnidaria venoms are known to have paralytic, neurotoxic, cytotoxic, dermotoxic, and hemolytic effects (Mariottini and Pane 2010).

Cancer is a very important public health problem which is seriously increasing in the World. One in every six deaths is caused by cancer (WHO 2020). In Türkiye, cancer is the second cause of death after cardiovascular illnesses (TÜİK 2023). According to World Health Organization data, the cancer types with the highest incidence are breast cancer (46.82%), prostate cancer (29.42%), lung cancer (23.62%), colorectal cancer (18.42%) and cervical cancer (14.12%). Lung and prostate cancers are the most common cancers in men; breast and lung cancers are the most common cancers in women (WHO/IARC 2024). According to 2022 data, the top 5 cancer types with the highest incidence in Türkiye are breast cancer (46.82), lung cancer (37.92), prostate cancer (35.32), colorectal cancer (19.82), and thyroid cancer (15.62), respectively. When looking at the distribution of cancer types by gender, the most common cancer types in women are breast, thyroid, uterine, and lung cancer; and in men, lung, prostate, colorectal, bladder, and stomach cancer (WHO/IARC 2024).

In recent years, many studies were done related on pharmacologic potentials, anti-inflammatory, anti-arrhythmic activities, antihypertensive, antimicrobial, analgesic, anticoagulant, antioxidant, anticancer and antitumor activities of Cnidaria venoms (Table 1).

**Table 1:** Various effects of venoms of Cnidaria species

Species	Venom Effect	References
*Anemonia viridis*	Cytotoxic	Bulati et al. 2016
*Anemonia sulcata*	Antiproliferative effect	Silva et al. 2017
*Anemonia sulcata*	Cytotoxic	Peña et al. 2023
*Anemonia sulcata*	Antimicrobial activity	Trapani et al. 2014
*Anemonia sulcata*	Anti-inflamatuar etki	Silva et al. 2017
*Anemonia sulcata*	Anti-inflammatory effect and immunomodulatory activities	Aassila et al. 2013
*Anemonia sulcata*	Cytotoxic	Carli et al. 1996
*Aurelia aurita*	Anticoagulant effect	Rastogi et al. 2012
*Chrysaora quinquecirrha*	Antitumor and antioxidant effect	Krishnan et al. 2014 Balamurugan et al. 2010
*Nemopilema nomurai*	Antioxidant and anticancer effect (liver cancer)	Choudhary et al. 2018
*Aequorea victoria*	Anticancer effect (prostate cancer, pancreatic cancer, colon cancer)	Katz et al. 2003 Rose et al. 2006
*Rhopilema nomadica*	Anticancer effect (liver cancer)	Tawfik et al. 2021
*Acromitus flagellatus*	Anticancer effect (breast cancer)	Hemavathi et al. 2014
*Rhizostoma octopus*	Cytotoxic, antitümör etki	Salama et al. 2022
*Pelagia noctiluca*	Anticancer effect (colon cancer)	Ayed et al. 2011
*Cassiopea andromeda*	Anticancer effect (breast cancer)	Mirshamsi et al. 2017
*Rhopilema esculentum*	Antihypertensive effect	Zhuang et al. 2012
*Chrysaora quinquecirrha*	Antimicrobial effect	Suganthi et al. 2012
*Cyanea capillata*	Antibacterial, antifungal effect	Zhou et al. 2018
*Porpita porpita*	Antibacterial, antifungal effect	Umamageswari et al. 2016
*Pelagia noctiluca*	Analgesic effect	Ayed et al. 2012
*Rhizostoma pulmo*	Anticoagulant	Rastogi et al. 2017
*Velella velella*	Cytotoxic (mouse lung fibroblasts)	Killi et al. 2020
*Aurelia aurita*	Anticancer effect (colorectal cancer)	Mazlum et al. 2026

In this study, it was targeted to obtain data for the pharmacologically potential of Cnidarians which threat the ecosystem function (Affecting fish population by directly consuming fish eggs and larvae and indirectly consuming zooplankton, contributing to form musilage), human health (negatively affect to contact holidaymakers causes pain, ache, burning, itching, redness, edema and blister formation) but are not evaluated economically. And also, with this research, for the first time in Türkiye, venom obtained from an anemone species has been demonstrated to be a usable alternative source in cancer research.

## Materials and Methods

### Sampling

In this study, individuals of *Aurelia aurita*, *Anemonia sulcata*, and *Rhopilema nomadica* were sampled. Thirteen individuals of *A. aurita* were sampled in November 2023 and 18 individuals in February 2024 in Gökova Bay with the direct observation method by hand net. *A. aurita* samples were put into plastic bags (50 × 30 × 20 cm) and then were transferred to the Marine Biology Laboratory of the Faculty of Fisheries. Fifteen *A. sulcata* individuals were sampled from the coastal zone of the Aquaculture Research and Development Unit of the Faculty of Fisheries, Ören, Milas, Muğla in May, 2023. Nine *R. nomadica* samples were obtained from trawl and purse seine fishermen in Mersin Bay in March 2024. The samples were then brought to Muğla Sıtkı Koçman University Faculty of Fisheries via cold chain. In venom isolation, the mouthparts of *A. aurita* and *R. nomadica* specimens and the tentacles of *A. sulcata* specimens were evaluated. The mouth arms and tentacles of the all samples were separated and stored in a deep freezer at -18 °C.

### Venom Isolation

Venom isolations were conducted according to Bloom et al. (1998) and Mariottini et al. (2002). Freezed tentacles and oral arms were defreezed in strained sea water at + 4 °C. It was provided to separate nematocysts from the epidermis by shaking over a night on the magnetic shaker., The samples put into the ice with water for this procedure was done + 4 °C. Then, the samples were filtered through 100 micrometer plankton net to remove the tissue residues. After that the samples was centrifuged with 4000 g at + 4 °C for 5 min. The nematocyst suspensions were counted on Sedgewick Rafter counting chamber and the nematocyst isolation continued to reach 200.000 nematocyst/ml. Then, the nematocyst suspensions were sonicated 90 times with the 30 s intervals on ice. The samples then were centrifuged again at 4000 g and + 4 °C for 5 min. Supernatants (venoms) were taken and stored at -18 °C. Protein concentrations were determined using the Bradford assay (Bradford 1976). Bovine serum albumin (BSA) was used as a standard to generate a calibration curve at concentrations of 5, 10, 15, 20, 40, 60, and 80 µg. The standards were prepared in cuvettes and the final volume was adjusted to 2 mL with Bradford reagent. For the venom samples, 5 µL of each isolated venom solution was mixed with 1995 µL of Bradford reagent and incubated at room temperature for 25–30 min. Absorbance was measured at 595 nm using a spectrophotometer, and protein concentrations were calculated using the equation obtained from the standard curve.

### Cell Cultures

To determine the cytotoxic effect of venom, two different colon cancer cell lines (HT29 and Caco2) and the healthy colon cell line CCD18co as a control were used. Cells were cultured in Dulbecco’s modified Eagle’s medium (DMEM) enriched with penicillin G (100 U/ml), streptomycin (100 µg/ml), L-glutamine, and 10% heat-inactivated fetal bovine serum (FBS) in an oven at 37 °C, 5% CO2, and 95% humidity.

### MTT Cytotoxicity Tests

Cytotoxic doses of venoms were determined by the MTT (3-(4,5-dimethylthiazol-2-yl)-2,5-diphenyltetrazolium bromide) assay. MTT measurement is a quantitative colorimetric method used to measure cytotoxicity based on the viability of metabolism in vitro. For the MTT assay, HT29 and Caco2 colon cancer cell lines and CCD18co healthy colon cell line were seeded in 96-well plates at 5 × 103 cells/well (3 replicates) in 200 µl of medium. After 24 h, cells were treated with different doses of venom (*A. aurita* and *R. nomadica *3.9–250 µg/ml; *A. sulcata* 0,93 − 6000 µg/ml). At the end of 24, 48 and 72 h of incubation, 20 µl of MTT was added to each well and incubated for 3 h at 37 °C in an incubator. At the end of the period, 100 µl of DMSO was added to the wells, and the color intensity after 20 min was measured at 540 nm using an ELISA Plate Reader. Using these absorbance values, comparative analysis was performed with the control group, and the concentrations causing 50% cell death (IC50) and cell viability percentages for each venom were calculated (Sakallı Çetin et al. 2017).

### Annexin V-7AAD Staining

In this study, cells undergoing apoptosis were determined using flow cytometry using Annexin V-7AAD. For this purpose, HT29 and Caco2 colon cancer cell lines were seeded in 6-well plates with 2 × 105 cells per well in 3 mL of medium. After 24 h, cells were treated with venom at IC50 doses (in triplicate). After 24 h of incubation, control and treated cells were placed in separate falcon tubes and centrifuged twice with PBS. After centrifugation, the cell pellet was treated with 1X binding buffer to reach 1 × 106 cells/ml. 100 µL of this cell solution was added, 5 µL of annexin V-PE and 5 µL of 7AAD-PERC were added, and incubated for 15 min at room temperature in the dark. 400 µl of 1X binding buffer was added to perform flow cytometry measurements. Experiments were performed in technical triplicate.

### Mitochondrial Membrane Potential Assay (MMP)

Changes in mitochondrial membrane potential (MMP) in the early stages of cell apoptosis were determined using a mitochondrial membrane potential determination kit. For this purpose, HT29 and Caco2 colon cancer cell lines were seeded in 6-well plates with 2 × 105 cells in 3 mL of medium per well. After 24 h, cells were treated with venom at IC50 doses. After 24 h of incubation, control and treated cells were placed in separate falcon tubes and centrifuged twice with PBS. The cells were incubated with TMRE at 37 °C, 5% CO2. At the end of the 24-hour incubation period, the cells were centrifuged, transferred to 3 ml of PBS, and measurements were performed by flow cytometry at FITC 575 nm. All experiments were performed in triplicate (Sakallı Çetin et al. 2017).

### Measurements of Reactive Oxygene Species (ROS)

Reactive Oxygen Species (ROS) formed in cells were determined by flow cytometry using a ROS measurement kit. For this purpose, cancer cell lines were seeded in 6-well plates with 2 × 10^5^ cells per well in 3 mL of medium and incubated for 24 h at 37 °C in a 5% CO2 incubator. Cells were incubated with 100 µL of ROS Assay Stain Solution for 60 min and then treated with IC50 doses of venom. At the end of this period, measurements were performed by flow cytometry at FITC 488 nm using a ROS measurement kit according to the kit’s procedure. All experiments were performed in triplicate (Sakallı Çetin et al. 2017).

### Statistical Analysis

All statistical analyses and graphical representations were performed using GraphPad Prism version 10 software (GraphPad Software, Boston, MA, USA). Quantitative data obtained from independent experiments, which were performed in triplicate, are presented as the mean ± standard deviation (SD).

To evaluate cell viability, the half-maximal inhibitory concentration IC_50_ values of the venoms were determined through nonlinear regression analysis. Data were fitted to a full sigmoidal dose-response curve utilizing the “[Inhibitor] vs. normalized response -- Variable slope” mathematical model. The robustness of the curve fitting was validated by evaluating the goodness-of-fit (R^2^ > 0.95) and establishing the 95% profile likelihood confidence intervals for each parameter.

For the comparative evaluation of flow cytometry data—including Annexin V/7-AAD apoptosis percentages, Reactive Oxygen Species (ROS) generation, and Mitochondrial Membrane Potential (MMP) variations—statistical significance among the experimental groups was assessed using a One-Way Analysis of Variance (ANOVA). Following the ANOVA, Dunnett’s multiple comparisons post-hoc test was applied to determine specific differences between individual treatment groups and the untreated control. Differences were considered statistically significant at a level of *p* < 0.05.

## Results and Discussion

The protein concentrations of ***Anemonia sulcata***, ***Aurelia aurita***, and ***Rhopilema nomadica*** venoms were 9.89 mg/mL, 0.70 mg/mL, and 2.73 mg/mL, respectively. Based on these values, the initial concentrations used in the MTT assay were 6000 µg /mL, 250 µg/mL, and 250 µg/mL, respectively. Serial dilutions were prepared at a 1:1 ratio. IC50 values ​​representing the dose causing 50% cell death using MTT assay results after 24, 48 and 72 h of exposure of cells to venom of all three species are shown in Table 2.

**Table 2:** IC50 values ​​determined using MTT assays

Species	Time (Hour)	CCD18co	Caco2	HT29
*Aurelia aurita *(µg/ml)	24	30.33 ± 0.41	30.81 ± 0.77	28.46 ± 0.09
	48	29.00 ± 0.78	24.48 ± 0.65	17.83 ± 0.05
	72	24.01 ± 0.71	15.11 ± 0.57	17.62 ± 0.03
*Rhopilema nomadica *(µg/ml)	24	34.07 ± 0.05	27.55 ± 0.53	42.45 ± 0.05
	48	33.54 ± 0.13	26.46 ± 0.24	41.76 ± 0.01
	72	18.68 ± 0.02	18.79 ± 0.18	35.27 ± 0.13
*Anemonia sulcata *(µg/ml)	24	6000 >	6000 >	6000>
	48	5858 ± 2.10	5673 ± 3.56	3269 ± 1.05
	72	1221 ± 2.3	731 ± 1.05	791 ± 0.8

**Fig. 1:**
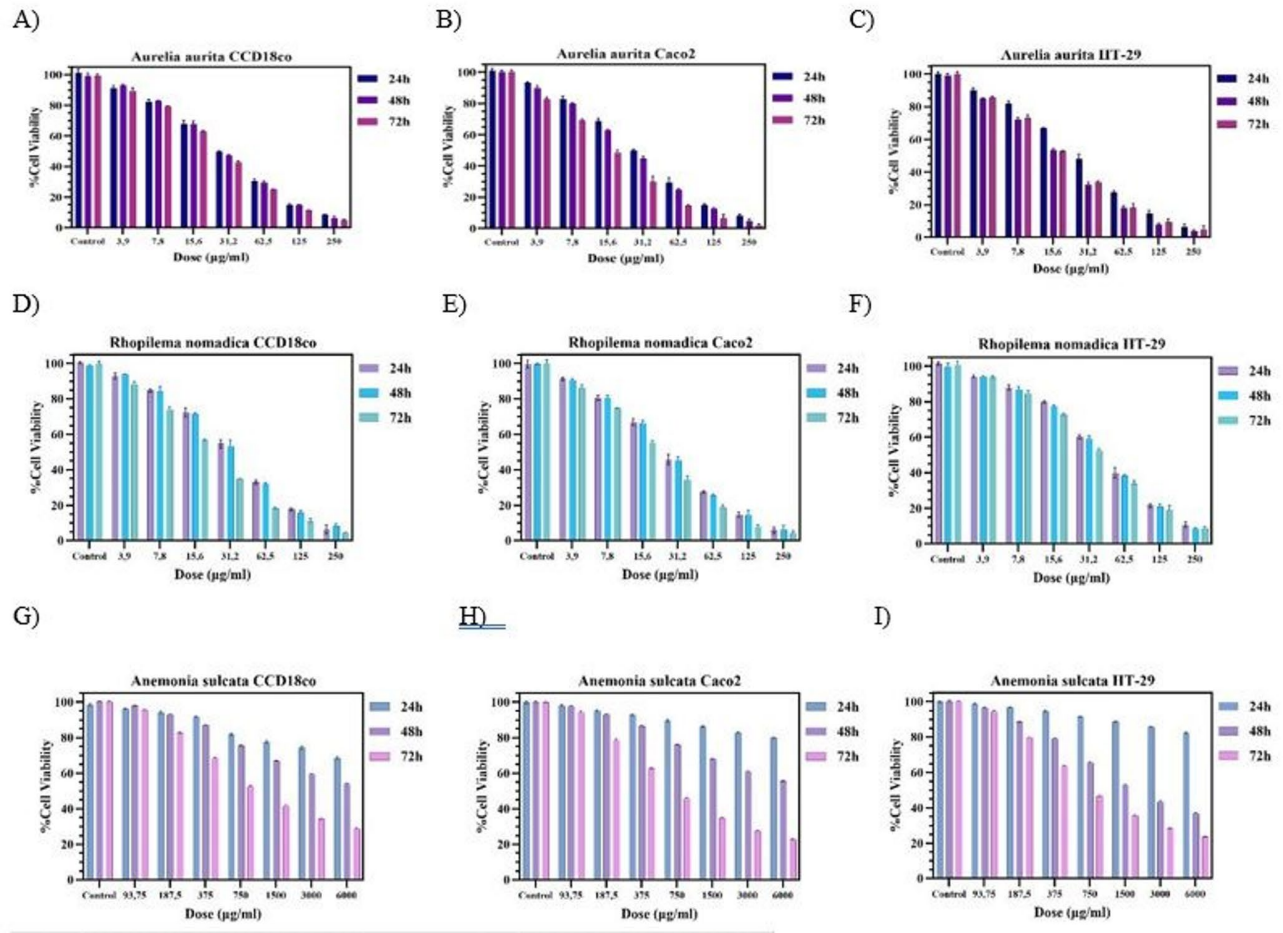
Dose-response profiles of *Aurelia aurita* (**A-C**), *Rhopilema nomadica* (**D-F**), and *Anemonia sulcata* (**G-I**) venoms on CCD18Co, Caco2, and HT29 cell lines after 24, 48, and 72 h exposure as determined by the MTT assay. Cell viability is expressed as % of control across increasing venom concentrations. Error bars indicate measurement variability

Across all panels, the MTT assay revealed a general dose-dependent decrease in cell viability, with cytotoxicity becoming more pronounced with longer exposure times (Fig. 1). *A. aurita* exhibited stronger cytotoxicity to both cancer cell lines than in the healthy cell line and remained effective at lower concentrations. *A. aurita* demonstrated greater cytotoxic potency in HT29 cells, with IC₅₀ values of 28.46 ± 0.09, 17.83 ± 0.05, and 17.62 ± 0.03 µg/mL at 24, 48, and 72 h, respectively, compared with higher IC₅₀ values in Caco2 cells (30,81 ± 0,77, 24,48 ± 0,65 and 15,11 ± 0,57 µg/mL at the assessed time points).

The MTT results showed that *R. nomadica* exerted a stronger inhibitory effect on the tested cancer cell lines than on the healthy cell line, in a dose-dependent manner, particularly in Caco2 cells. For *R. nomadica*, time-dependent sensitization was evident in Caco2 (72 h IC₅₀: 18.79 ± 0.18 µg/mL), whereas HT29 remained comparatively more resistant (72 h IC₅₀: 35.27 ± 0.13 µg/mL).

For *A. sulcata*, cytotoxicity was minimal at 24 h in all three cell lines, with IC₅₀ values remaining above the highest tested concentration (IC₅₀ > 6000 µg/mL; Table 2). At both 48 and 72 h, *A. sulcata* is more cytotoxic to both cancer cells than to healthy cells. At 48 h, sensitivity increased, most notably in HT29 (IC₅₀: 3269 ± 1.05 µg/mL /mL), and by 72 h IC₅₀ values markedly decreased in both cancer cell lines (Caco2: 731 ± 1.05 µg/mL; HT29: 791 ± 0.8 µg/mL).

Collectively, these findings indicate that venom-induced cytotoxicity varies both by venom species and by cell line. Based on IC₅₀ trends, *A. aurita* displayed the highest cytotoxic potency (low µg/mL IC₅₀ range), *R. nomadica* showed a moderate and cell line–dependent effect, and *A. sulcata* exhibited a delayed, high-dose cytotoxicity profile, becoming clearly evident at 72 h. Overall, the three venoms demonstrate distinct potency and temporal kinetics across the tested cell models. Therefore, to further characterize venom-induced cell death and associated mechanisms, Annexin V staining, mitochondrial membrane potential (MMP) assessment, and reactive oxygen species (ROS) measurements were performed in the more sensitive Caco2 (*R. nomadica*) and HT29 cells (*A. aurita* and *A. sulcata*) at 48 h.

### Selectivity Index (SI) Evaluation

To evaluate the specific antitumor potential of the tested venoms against cancer cells over healthy cells, the selectivity index (SI) was calculated for 24, 48, and 72-hour incubation periods. The SI is a widely used parameter to express the in vitro efficacy of a compound, and it was calculated according to the following equation:

$$\:SI=\frac{{IC}_{50\:}of\:healthy\:cells}{{IC}_{50}\:of\:cancer\:cells}$$

An SI value greater than 1 typically indicates that the test agent is more toxic to the tumor cell line than to the normal cells. As summarized in Table 3, the evaluated venoms demonstrated varying degrees of selectivity depending on the cell line and exposure time. Although the *Rhopilema nomadica* extract exhibited general toxicity, including in healthy cells, it still achieved SI > 1 against the Caco2 cell line at 24 and 48 h (1.23 and 1.26, respectively). Both *Aurelia aurita* and *Anemonia sulcata* demonstrated increasingly favorable SI values at 48 and 72 h, reaching up to 1.62 and 1.78 against the HT29 cell line, respectively.

**Table 3:** Selectivity Index (SI) values of *A. aurita*,* R. nomadica*, and *A. sulcata* venoms on Caco2 and HT29 cell lines across 24, 48, and 72 h

Time (h)	*A. aurita*		*R*. nomadica		*A. sulcata*	
	CCD18Co/ Caco2	CCD18Co/ HT29	CCD18Co/ Caco2	CCD18Co/ HT29	CCD18Co/ Caco2	CCD18Co/ HT29
24	0.98	1.06	1.23	0.80	-	-
48	1.18	1.62	1.26	0.80	1.03	1.78
72	1.58	1.36	0.99	0.52	1.67	1.54

*SI, cytotoxic selectivity index (the degree of selectivity between healty cells and cancer cells, expressed as SI = IC_50_ on normal cells/IC_50_ on cancer cells)

### Annexin V/7-AAD Staining Results

Induction of apoptosis has been used to assess the efficacy of venoms for inhibiting cancer cell proliferation. The venoms showed cytotoxic effects on cancer cells; therefore, flow cytometry analysis (*A. aurita* and *A. sulcata*; BD Accuri C6 Plus-Flow Cytometer, *R. nomadica*; Beckman Coulter Cytoflex) was performed to determine whether they promoted apoptosis.

**Fig. 2:**
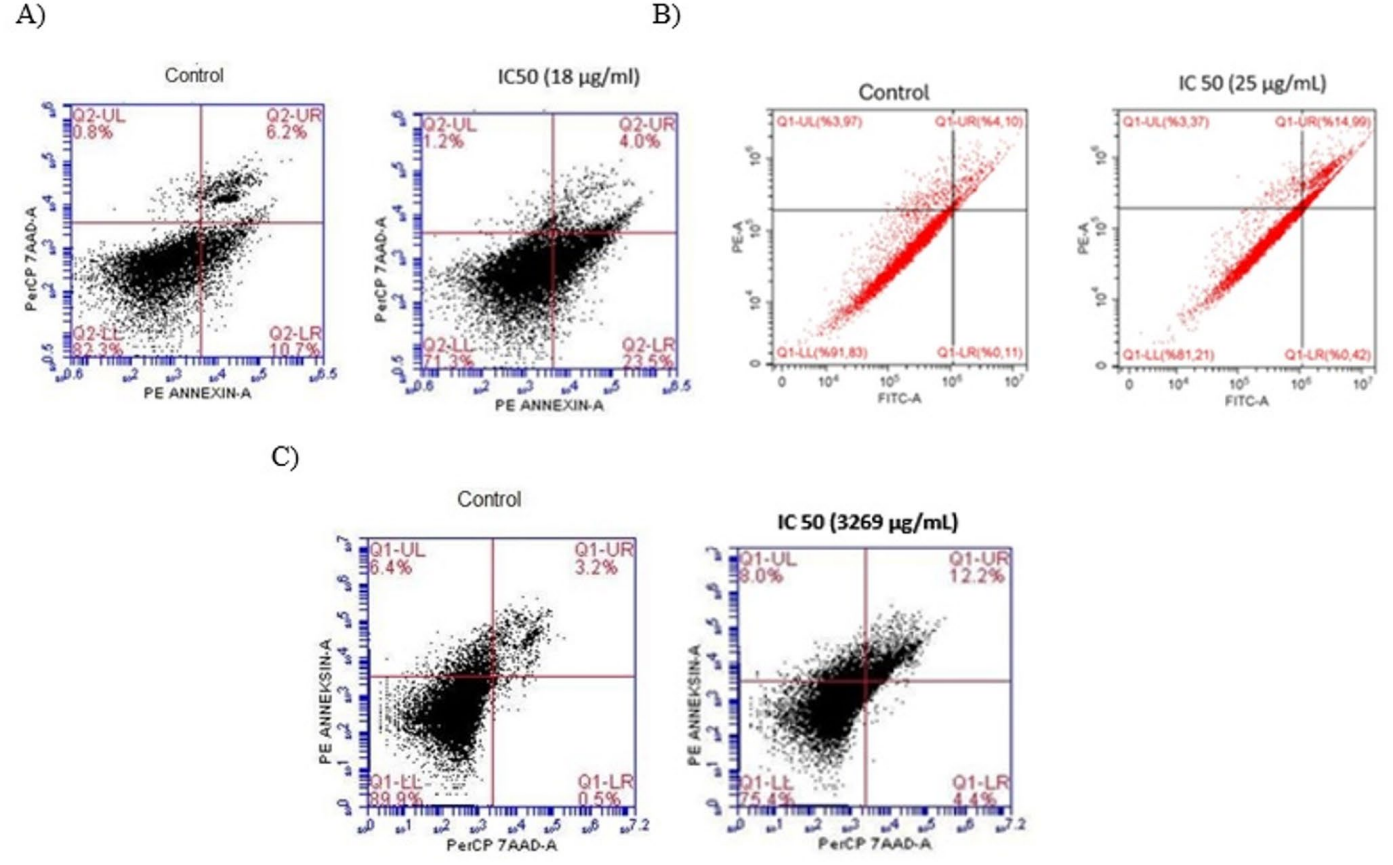
Apoptotic effects of venom treatments on HT29 and Caco2 cells at IC50 concentrations (*A. aurita*, 18 µg/mL; *A. sulcata*, 3269 µg/mL; *R. nomadica*, 25 µg/mL). (A) Comparative analysis of total apoptosis percentages between control and treatment groups (****p < 0.0001). (B) Fractional distribution of viable, total apoptotic, and necrotic cell populations following venom exposure

**Fig. 3:**
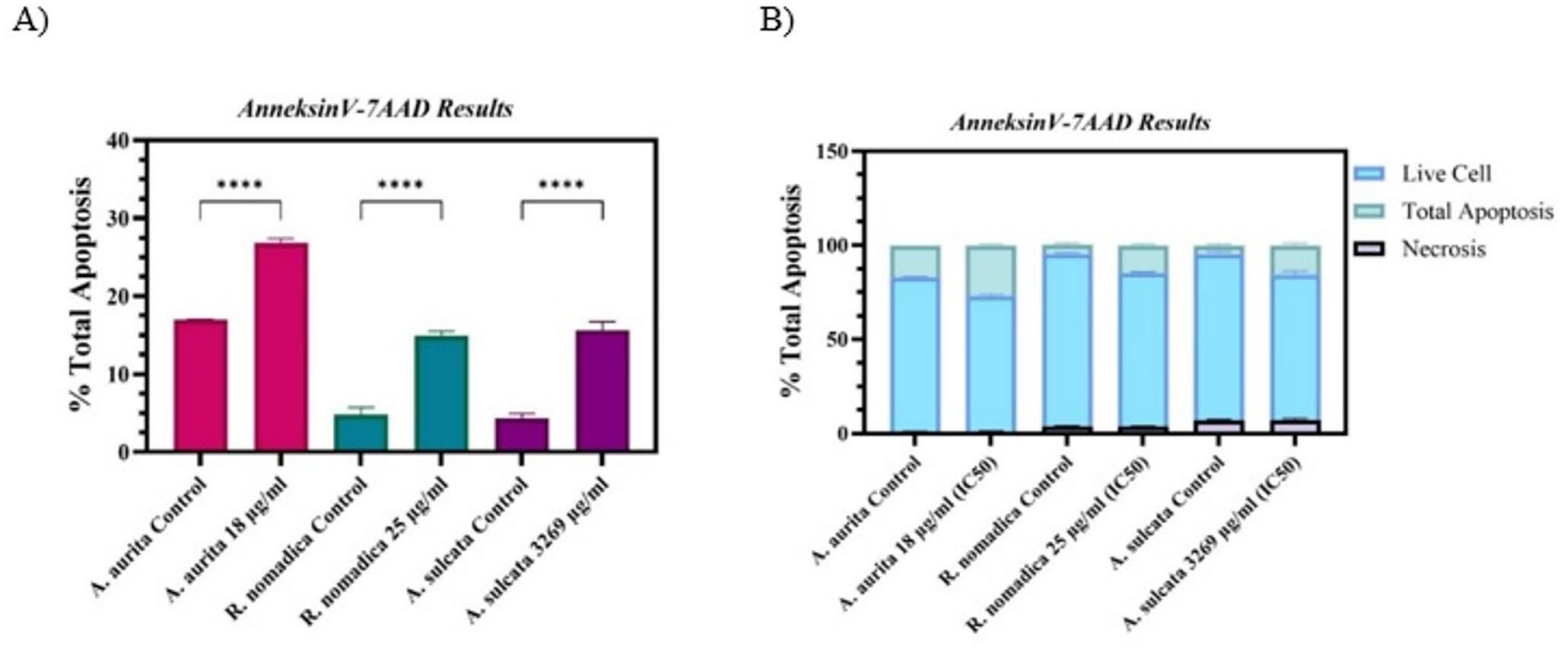
(**A**) Flow cytometry results from the apoptotic effect of *A. aurita* venom on HT29 cells. (**B**) Flow cytometry results of the apoptotic effect of *R. nomadica* venom on Caco2 cell (**C**) Flow cytometry results of the apoptotic effect of *A. sulcata* venom on HT29 cells

Given that *A. aurita*, *R. nomadica*, and *A. sulcata* venoms reduced cell viability and showed pronounced antitumoral activity in HT29 and Caco2 cells, Annexin V staining with flow cytometry was used to assess whether the cytotoxic effects were mediated by apoptosis. As shown in Figs. 3 and 2, treatment of HT29 cells with *A. aurita* venom at its IC₅₀ concentration (18 µg/mL) for 48 h increased the apoptotic cell population to 26.83%, compared with 16.96% in the control group. Similarly, exposure of Caco2 cells to *R. nomadica* venom at the IC₅₀ concentration (25 µg/mL) for 48 h resulted in an increase in apoptosis to 14.90%, whereas only 4.55% apoptotic cells were observed in the control group. Likewise, *A. sulcata* venom treatment at the IC₅₀ concentration (3269 µg/mL) markedly increased Annexin V–positive cells (16.6%) compared with untreated controls (3.7%), indicating enhanced apoptotic cell death. Overall, PE Annexin V/7-AAD analysis demonstrated that *A. aurita*, *R. nomadica*, and *A. sulcata* venoms at their IC₅₀ concentrations induce apoptosis in HT29 and Caco2 cells (Fig. 2).

Regarding the *Aurelia aurita* treatment, in the control cells, the mean of triplicate measurements revealed a viable cell rate of 80.37% (quadrant Q1-LL), 4.6% early apoptosis (quadrant Q1-LR), 8.5% late apoptosis (quadrant Q1-UR), and 6.57% necrosis (quadrant Q1-UL). The total apoptosis rate (early apoptosis + late apoptosis) in control HT29 cells was 13.10%. At the IC50 dose (18 µg/ml) of *Aurelia aurita* venom, the mean of triplicates showed a viable cell rate of 72.7% (quadrant Q1-LL), 5.97% early apoptosis (quadrant Q1-LR), 13.27% late apoptosis (quadrant Q1-UR), and 8.1% necrosis (quadrant Q1-UL). The total apoptosis rate in HT29 cells treated with 18 µg/ml venom was 19.17%. It was determined that the percentage of apoptosis significantly increased at the applied dose of *A. aurita* venom compared to the control (Fig. 3A).

Similarly, in the *R. nomadica* evaluation, the mean of triplicate measurements in the control cells demonstrated a viable cell rate of 91.60% (quadrant Q1-LL), 0.11% early apoptosis (quadrant Q1-LR), 4.44% late apoptosis (quadrant Q1-UR), and 3.85% necrosis (quadrant Q1-UL). The total apoptosis rate (early apoptosis + late apoptosis) in control CaCo2 cells was 4.55%. At the IC50 dose (25 µg/ml) of the venom, the mean of triplicates indicated a viable cell rate of 81.53% (quadrant Q1-LL), 0.4% early apoptosis (quadrant Q1-LR), 14.51% late apoptosis (quadrant Q1-UR), and 3.56% necrosis (quadrant Q1-UL). The total apoptosis rate in CaCo2 cells treated with 25 µg/ml venom was 14.91% (Fig. 3B).

Furthermore, regarding the *A. sulcata* application, the mean of triplicate measurements for the control cells showed a viable cell rate of 88.63% (quadrant Q1-LL), 0.6% early apoptosis (quadrant Q1-LR), 3.7% late apoptosis (quadrant Q1-UR), and 6.96% necrosis (quadrant Q1-UL). The total apoptosis rate (early apoptosis + late apoptosis) in control HT29 cells was 4.37%. At the IC50 dose (3269 µg/ml) of the venom, the mean of triplicates revealed a viable cell rate of 77.13% (quadrant Q1-LL), 4.84% early apoptosis (quadrant Q1-LR), 10.8% late apoptosis (quadrant Q1-UR), and 7.2% necrosis (quadrant Q1-UL). The total apoptosis rate in HT29 cells treated with 3269 µg/ml venom was 15.63% (Fig. 3C).

To evaluate the mechanisms of cell death induced by the venom treatments, Annexin V/7-AAD double staining was performed and analyzed via flow cytometry. As illustrated in Fig. 2A, treatment with the IC50 concentrations of *Aurelia aurita* (18 µg/mL), *Rhopilema nomadica* (25 µg/mL), and *Anemonia sulcata* (3269 µg/mL) venoms resulted in a highly significant increase in the total apoptosis percentage compared to their respective untreated control groups (p < 0.0001). Furthermore, the cell population distribution analysis presented in Fig. 2B revealed that the cytotoxic effect of the venoms was primarily mediated through apoptotic pathways rather than non-specific necrosis; this is evidenced by the marked reduction in the viable cell fraction and a concurrent expansion of the total apoptosis fraction, while the percentage of necrotic cells remained notably minimal across all experimental groups.

### MMP Assay Results

**Fig. 4:**
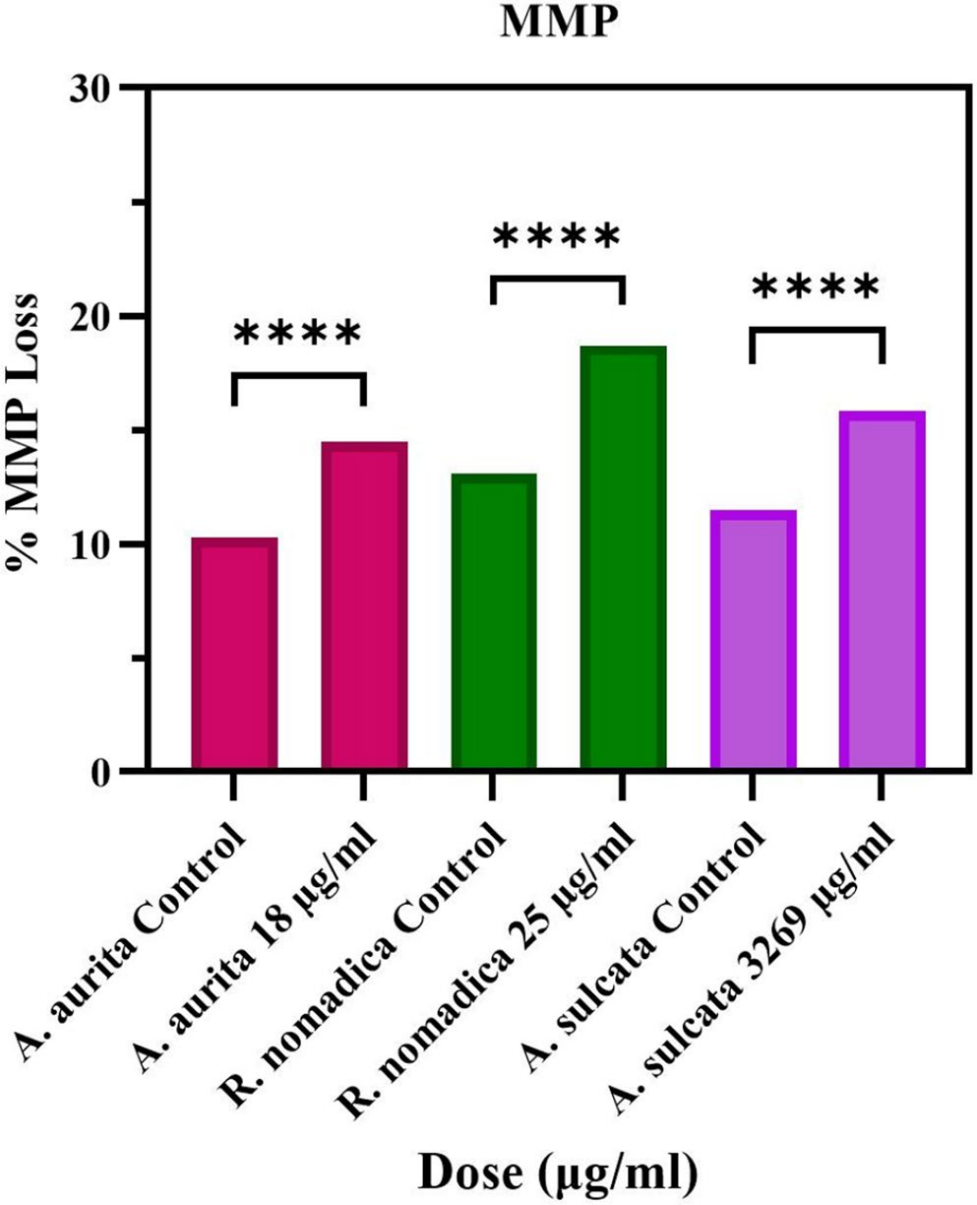
Mitochondrial membrane potential (MMP) loss (%) in HT29 and Caco2 cells after venom treatment at the IC₅₀ concentrations (*A. aurita* 18 µg/mL; *A. sulcata* 3269 µg/mL; *R. nomadica* 25 µg/mL). (****p < 0.0001)

Mitochondrial dysfunction is a critical event in apoptosis. Accordingly, mitochondrial membrane potential (MMP) loss was evaluated after 48 h of venom exposure in the responsive cell models. In HT29 cells, treatment with *A. aurita* venom at its IC₅₀ concentration (18 µg/mL) resulted in increased MMP loss compared with the control group (Fig. 4). A similar increase in MMP loss was observed in HT29 cells following exposure to *A. sulcata* venom at the IC₅₀ concentration (3269 µg/mL) (Fig. 4). In Caco2 cells, *R. nomadica* venom treatment at the IC₅₀ concentration (25 µg/mL) also significantly enhanced MMP loss relative to controls (Fig. 4). Collectively, these findings demonstrate that all three venoms induce mitochondrial depolarization at their IC₅₀ concentrations under the experimental conditions (Fig. 4).

### ROS Measurements Results

**Fig. 5:**
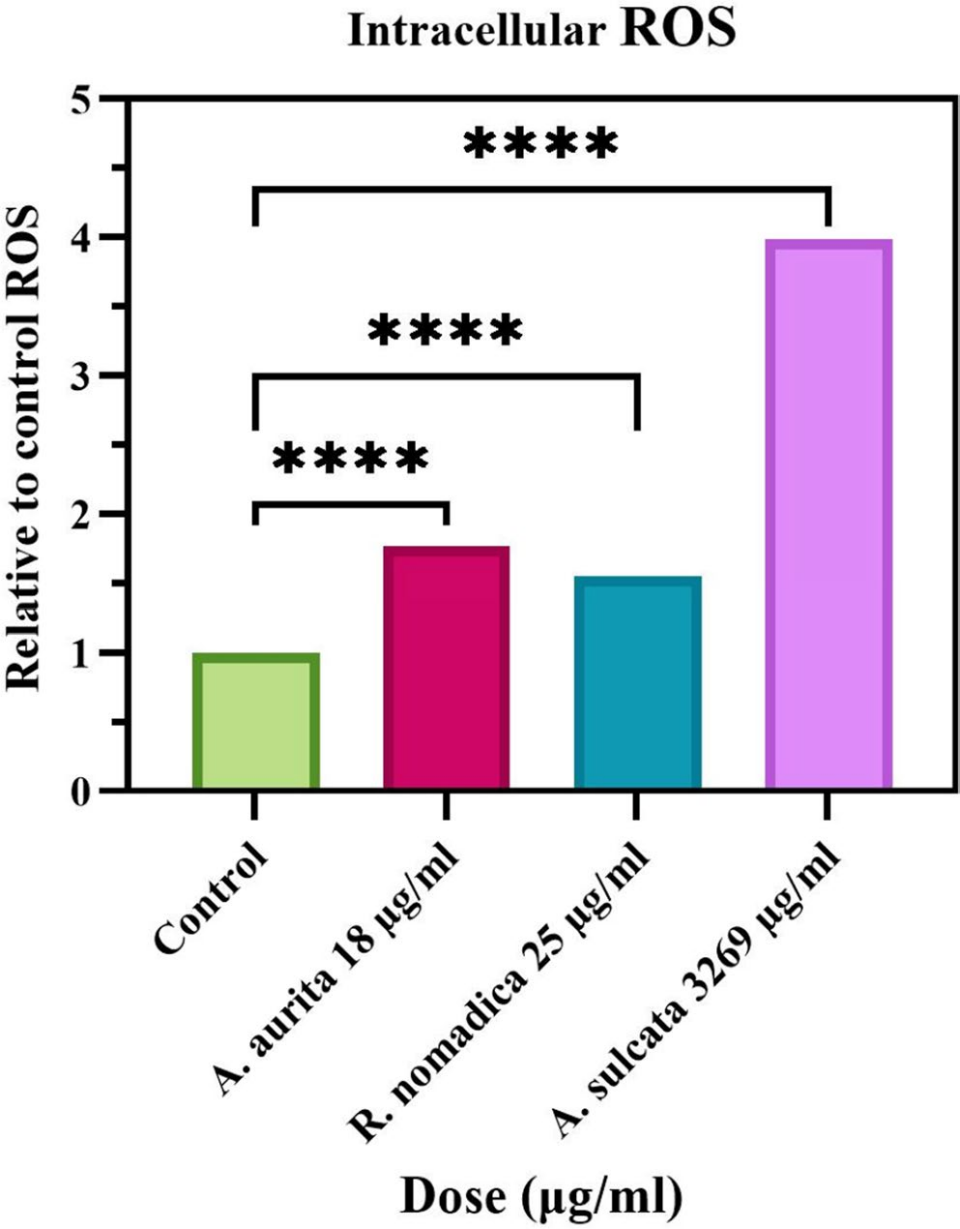
Intracellular ROS levels were measured by flow cytometry, normalized to the control. At the IC₅₀ concentrations, *A. aurita* (18 µg/mL), *A. sulcata* (3269 µg/mL), and *R. nomadica* (25 µg/mL) significantly increased relative ROS compared with the control (****p < 0.0001)

Intracellular reactive oxygen species (ROS) generation is closely linked to apoptosis. Accordingly, ROS levels were assessed after 48 h of venom exposure in the responsive cell models (Fig. 5). In HT29 cells, treatment with *A. aurita* venom increased ROS levels from 11.8% in the control group to 20.8% at the IC₅₀ concentration (18 µg/mL) (Fig. 5). Similarly, *R. nomadica* venom treatment elevated ROS levels in Caco2 cells from 3.06% in controls to a mean value of 4.7% at the IC₅₀ concentration (25 µg/mL) (Fig. 5). Likewise, exposure of HT29 cells to *A. sulcata* venom resulted in a marked increase in ROS production, rising from 8.2% in control cells to 32.7% at the IC₅₀ concentration (3269 µg/mL) (Fig. 5). Collectively, the enhanced fluorescence signal following venom treatment indicates increased ROS generation at IC₅₀ concentrations under the experimental conditions (Fig. 5).

These findings demonstrate that venoms from all three cnidarian species exert cytotoxic effects in two different colon cancer cell lines, and the observed cytotoxicity is supported by apoptosis-related readouts. *A. aurita* venom caused high MMP loss and increased apoptosis in HT29 cells, but did not show a dominant increase in ROS. *R. nomadica* venom caused high ROS in Caco2 cells but had a low effect on MMP loss. *A. sulcata* venom, however, can be said to have shown a slow but consistent effect, especially when Caco2 and HT29 cells are considered together, causing high ROS, significant MMP loss, and increased apoptosis.

Although several studies have examined the cytotoxic effects of *Anemonia sulcata* venom (see Table 1), our study is the first to evaluate its effects in two different colon cancer cell lines. In addition, to the best of our knowledge, no previous study has evaluated the effects of *Aurelia aurita* and *Rhopilema nomadica* venoms in colon cancer models.

In this study, crude venom was used because the biological activity of cnidarian venoms often results from synergistic interactions among multiple toxin components. Previous studies have shown that isolated toxin peptides may exhibit lower cytotoxic activity compared with crude venom preparations (Nagai 2003).

In the study by Silva et al. (2017), it was observed that the *A. sulcata* extract became more toxic over time and caused a 62.63% decrease in cell viability after 72 h at the highest concentration tested (1 mg/mL). Similarly, in the study by Killi et al. (2020), it was observed that the crude venom of *Velella vellella* was cytotoxic in L929 cells at high doses, while at low doses it increased cell proliferation and metabolic activity. In parallel with these studies, the fact that the cytotoxic effect of *A. sulcata* venom was more pronounced at the highest doses in our study can be explained by the dose-dependent effect of the venom on the cells. A large portion of sea anemone venoms affect Na + and K+ channels, altering the inactivation kinetics of these channels and disrupting ion balance (Honma and Shiomi 2006; Moran et al. 2012). While this ion imbalance can be compensated for at low doses, it is rapidly disrupted at high doses. In particular, Ca2 + accumulation irreversibly triggers cellular stress responses (Piccialli et al. 2021). Furthermore, high-dose exposure increases the production of ROS species, triggering DNA damage and endoplasmic reticulum (ER) stress, and even leading to changes in membrane permeability or pore formation (Hetz 2012; Honma and Shiomi 2006).

In contrast, the more pronounced MMP loss caused by *Aurelia aurita* venom in HT29 cells indicates that this venom directly targets mitochondria. The lower MMP loss caused by *Anemonia* venom in the same cell line, despite higher ROS production, suggests that oxidative stress alone is not sufficient for mitochondrial damage and that HT29 cells may be more resistant to mitochondrial damage.

Despite the high cytotoxicity of *Aurelia aurita* venom, the moderate level of ROS-induced apoptosis suggests that it causes cell death through different mechanisms. Bayazit’s (2004) study showed that enzymes obtained from *A. aurita* extract affect different phases of the mitotic cycle in cancer cells and lead to the breaking of ester bonds in DNA. Therefore, it is thought that in our study, in addition to ROS-induced apoptosis, different mechanisms are involved.

In a study by Tawfik et al. (2021), R. *nomadica* venom showed significant dose-dependent cytotoxicity in HepG2 (hepatocellular carcinoma) cells after 48 h of treatment with an IC50 value of 50 µg/mL. It was determined that *R. nomadica* venom caused a significant increase in apoptosis. These findings indicate that *R. nomadica* venom induces apoptosis in hepatocellular carcinoma cells (Tawfik et al. 2021). In our study, *R. nomadica* venom was found to have a cytotoxic effect, triggering apoptosis in the Caco2 cell line over 48 h. Furthermore, Annexin-V, MMP, and ROS analyses revealed that apoptosis increased in cells exposed to twice the amount of R. nomadica venom. The fact that *Rhopilema nomadica* venom exhibits limited MMP effect despite high ROS production, particularly in CaCO2 cells, suggests that its cytotoxicity does not cause mitochondrial collapse, but rather that other mechanisms (e.g., protein degradation, disruption of cellular balance) are at play. When these data are considered together, it becomes clear that the cytotoxic effects of venoms cannot be explained by a single pathway; rather, a multi-component mechanism is involved, with ion channel modulation, oxidative stress, ER stress, and mitochondrial dysfunction contributing to varying degrees depending on the cell type.

In a study conducted by Salama et al. (2022), *Rhizostoma octopus* venom extract was tested on hepatocellular carcinoma (HepG-2, liver cancer) and breast (MCF-7) cancer lines, and the 24-hour IC50 values were found to be 808.4 µg/ml and 896.4 µg/ml, respectively. The chemotherapy drug Doxorubicin (Dox) was used as a control group, and the IC50 value of Dox was found to be 0.37 µg/ml in HepG-2 and 0.36 µg/ml in MCF-7. In other words, it was determined that *R. octopus* venom was not as effective as the chemotherapy drug in killing cancer cells. When given in high doses and for long periods, chemotherapy drugs increase necrosis in both cancerous and healthy cells (Elmore 2007). Apoptosis kills cells by fragmenting them, while necrosis disrupts the cell membrane, causing the dispersion of cell contents and, consequently, inflammation. Inflammation is undesirable for cancer patients because it exacerbates the side effects of chemotherapy. Cancer drugs generally attempt to kill cells through the induction of apoptosis (Elmore 2007). Our study also determined that the cytotoxic effects of venoms from three species are mediated by apoptosis, and necrosis rates are low.

In another study by Ayed et al. (2011), *Pelagia noctiluca* raw venom was applied to the HCT116 (human colon cancer) line using the MTT method and incubated for 24 h. It was determined that P. noctiluca crude venom caused cell death in a dose-dependent manner, with an IC50 value of 320 µg/ml at the end of 24 h. Cancer cell death was reported to occur due to DNA fragmentation and oxidative stress.

In a study by Krishnan et al. (2014) and Dehghani et al. (2021), *Cassiopea andromeda* venom was applied to the NB4 cell line, a type of white blood cell cancer called acute promyelocytic leukemia. Cell viability was measured at 24, 48, and 72-hour intervals using the MTT method. Venom was found to be more effective after 72 h compared to other time series. It was determined that venom inhibited the proliferation of NB4 cells by increasing apoptosis, arrested the cell cycle, and reduced cell number by preventing uncontrolled proliferation (Dehghani et al. 2021). The cytotoxic effects of the *A. aurita*, *R. nomadica*, and *A. sulcata* venoms used in our study were observed to be optimal at 48 h. The findings indicate that the cellular effects of different venoms depend not only on the nature of the toxin but also on cell type-specific response mechanisms. Although *Anemonia sulcata* venom causes high MMP loss in CaCO2 cells, the fact that this effect occurs at high doses and with a delayed time frame of 48–72 h suggests that the toxin does not directly target mitochondria, but rather leads to indirect mitochondrial disruption due to channel permeability, Ca²⁺ imbalance, and endoplasmic reticulum (ER) stress (Hetz 2012; Sano and Reed 2013). Indeed, studies with sea anemone extracts have shown that ROS levels can be modulated and antioxidant/detoxifying systems can also be activated (Silva et al. 2017; Peña et al. 2023). This suggests that when oxidative stress in cells remains below a certain threshold, it triggers adaptive responses rather than directly leading to cytotoxicity. Specifically, the “unfolded protein response” (UPR) and antioxidant enzyme systems (e.g., GPX, GST) may play a protective role in this phase (Piccialli et al. 2020). Therefore, although the observed increase in ROS indicates the onset of cell damage, it can be said that this increase alone is not sufficient to kill 50% of the cell population, and thus the IC50 values ​​remain high.

In a study conducted with *Cyanea nozakii* venom, cytotoxic activity was observed in H630 colon cancer cell lines treated with an IC50 dose of 5.1 µg/ml for 48 h. The venom was found to cause cell death by damaging the cell membrane (Cuiping et al. 2012). *Nemopilema nomuraii* venom was tested on HepG2 (hepatocellular carcinoma), MDA-MB231 (breast cancer), HT29 (colon cancer), and A549 (lung cancer) cancer lines, and was noted to exhibit the most cytotoxic effect in the HepG2 cancer line (Lee et al. 2017). Considering this literature, the cytotoxic effect of Cnidaria venoms appears to be selective in different cancer lines. In this study, *A. aurita* and *A. sulcata* from three different species showed cytotoxic effects on the HT29 cell line, while *R. nomadica* venom was effective on the Caco2 cell line. Therefore, it appears that the venom of each species exhibits selectivity on different cells.

The selectivity index (SI) indicates the effect of venoms on cancer cells compared to healthy cells. Low IC50 values ​​indicate high cytotoxicity, while high SI values ​​indicate that a higher dose is required to kill healthy cells. An SI value close to 1 does not indicate significant selectivity. This may be due to experimental differences or malfunctions. For *R. nomadica*, the SI value is greater than 1, but the difference in IC50 values ​​between healthy and cancer cells is very small, indicating that it is not biologically selective. In other words, *R. nomadica* venom can also affect healthy cells. *A. sulcata* venom causes delayed apoptosis, so its SI increases over time. *A. aurita* venom, despite directly affecting mitochondria, shows cell selectivity, and its SI increases over time.

## Conclusion

According to the MTT results, lower IC₅₀ values indicate higher cytotoxic potency at the tested exposure time. Accordingly, *A. aurita* venom showed the strongest cytotoxic effect on HT29 cells and only minimal cytotoxicity in healthy cells at 48 h. *A. sulcata* venom also exhibited marked cytotoxic effects on HT29 cells at 48 and 72 h, while showing limited toxicity in healthy cells. *R. nomadica* venom displayed cytotoxic activity in Caco2 cells at 48 h. When the three venoms were compared in the healthy CCD18Co colon cell line, *R. nomadica* venom exhibited the highest cytotoxic effect among the tested venoms.

The higher sensitivity of HT29 cells to the venom treatments, compared to Caco2, can be attributed to their undifferentiated state and lack of tight junction formations under standard culture conditions (Martínez-Maqueda et al. 2015). This contrasts directly with the highly polarized, barrier-forming enterocyte-like phenotype of differentiated Caco2 cells, which structurally restricts venom penetration (Sambuy et al. 2005).

To explore the mode of cell death underlying these effects, apoptosis and related endpoints were evaluated at the IC₅₀ concentrations. Annexin V/7-AAD analysis indicated an increased apoptotic cell population relative to the untreated control in venom-treated groups, supporting apoptosis as a major contributor to the observed cytotoxicity. Under the IC₅₀ conditions, necrotic populations remained comparatively low in the evaluated models.

Loss of mitochondrial membrane potential is a hallmark of apoptosis initiation and is often accompanied by increased intracellular reactive oxygen species (ROS). Consistent with this, MMP loss and elevated ROS signals were observed following IC₅₀ venom exposure, further supporting the involvement of apoptosis-associated mechanisms in the cytotoxic response. Collectively, these findings suggest that venoms from three cnidarian species exert cytotoxic effects in two colon cancer cell lines, with apoptosis-related pathways contributing to this outcome. Based on the literature, further studies are warranted to assess cnidarian venoms across additional cell lines within the same cancer type and to broaden the evaluation of venoms from different cnidarian species in colon cancer models.

## Data Availability

Data Availability StatementThe data supporting the findings of this study are available from the corresponding author upon reasonable request.
